# Valedictory

**Published:** 1860-09

**Authors:** Jos. P. Logan, W. F. Westmoreland


### EDITORIAL AND MISCELLANEOUS.

### Valedictory.

While it is with a sensation of relief from a great burden, that we announce the dissolution of our connection with the Atlanta Medical Journal, *as its Editors*, it is, at the same time, with regret that we take leave of what has been to us —with all its cares, anxieties and unrequited efforts—"a labor of love." The present number commences the Sixth Volume, and with it the labors of others, who will, we hope, manage it with more ability than has characterized it heretofore. The Journal has arrived at an age which is beyond infancy, and has (we have no hesitation in asserting) acquired a respectable reputation, and has furnished, along with more or less inferior matter, a large amount of useful information, from a great number of highly competent contributors.

We have not undertaken, as announced at an early period in the history of this periodical, to be an infallible judge of merit—to publish nothing but that which would be regarded as meritorious by the rule of a rigid criticism; we have, upon the contrary, published many articles which seemed to us to be unworthy the space they occupied, and which have doubtless been complained of by some of our readers, but in this acknowledgment we only mean to say that we have attempted to carry out the original design of the Journal—to furnish a medium of intercommunication between the Medical men of the Southern country, and especially between those of this region of country and others who would become specially interested in the success of the enterprise, as connected with the institution of which it was the organ, and through which the small as well as the large Doctors might be heard. We hope, then, that having carried out as we think, the object and design of the publication, the more discriminating and dainty reader has not been the less satisfied with the pure and refined material which has been contained in the Journal, simply because it has contained some of a baser sort.

We must, however, in conclusion, acknowledge our many short-comings, and confess that we have committed a multitude of errors, for which we beg forgiveness of the kind and liberal patrons who have sustained and often commended the *Journal* for merits which we could not perceive. Before writing the last word, allow us to commend the incoming Editor to the kind and respectful consideration of our readers, as zealous, competent and worthy, with the hope that he may fare no worse at their hands than we have done, and that the forbearance and ever-ready appreciation which they exercised toward us, may be better requited at the hands of our successor.

Farewell, kind and generous friends; we shall never forget thee.

JOS. P. LOGAN,
W. F. WESTMORELAND.

